# Preparation of PLGA microspheres loaded with niclosamide via microfluidic technology and their inhibition of Caco-2 cell activity *in vitro*

**DOI:** 10.3389/fchem.2023.1249293

**Published:** 2023-09-14

**Authors:** Yulei Tai, Menglun Tian, Yu Chen, Peijun You, Xiaojun Song, Bangting Xu, Cidong Duan, Dazhi Jin

**Affiliations:** ^1^ School Laboratory of Medicine, Hangzhou Medical College, Hangzhou, Zhejiang, China; ^2^ Laboratory of Biomarkers and In Vitro Diagnosis Translation of Zhejiang Province, Hangzhou, Zhejiang, China

**Keywords:** niclosamide, poly(lactic-co-glycolic acid), hyaluronic acid, microfluidic technology, cell activity

## Abstract

Niclosamide (NIC) is a multifunctional drug that regulates various signaling pathways and biological processes. It is widely used for the treatment of cancer, viral infections, and metabolic disorders. However, its low water solubility limits its efficacy. In this study, poly(lactic-co-glycolic acid) (PLGA) and hyaluronic acid (HA), which exhibit good biocompatibility, biodegradability, and non-immunogenicity, were conjugated with niclosamide to prepare PLGA-HA-niclosamide polymeric nanoparticles (NIC@PLGA-HA) using microfluidic technology. The obtained microspheres had a uniform size distribution, with an average mean size of 442.0 ± 18.8 nm and zeta potential of −25.4 ± 0.41 mV, indicating their stable dispersion in water. The drug-loading efficiency was 8.70%. The drug-loaded microspheres showed sustained release behavior at pH 7.4 and 5.0, but not at pH 2.0, and the drug release kinetics were described by a quasi-first-order kinetic equation. The effect of the drug-loaded microspheres on the proliferation of Caco-2 cells was detected using the MTT assay. Hydrophilic HA-modified NIC@PLGA-HA microspheres prepared via microfluidic technology increased the cellular uptake by Caco-2 cells. Compared to the same concentration of NIC, the NIC@PLGA-HA microspheres demonstrated a stronger inhibitory effect on Caco-2 cells owing to the combined effect of PLGA, HA, and NIC. Therefore, the pH-responsive NIC@PLGA-HA microspheres synthesized using microfluid technology increased the solubility of NIC and improved its biological activity, thus contributing to the demand for intestinal drug carriers.

## 1 Introduction

Niclosamide (NIC), also known as N-(2′-chloro-4′-nitrophenyl)-5-chlorosalicylamide, with the molecular formula C_13_H_8_Cl_2_N_2_O_4_, is a salicylamide derivative. It was approved by the United States Food and Drug Administration (FDA) in 1982 and is listed on the World Health Organization’s list of essential medicines (Lohiya and Katti, 2021). It is poorly soluble in water at room temperature and poorly absorbed by the intestinal tract, which allows it to maintain a high concentration in the gut. NIC exerts antiparasitic effects by destroying the protective substances used by parasites to evade host cell proteases, or by inhibiting Adenosine Triphosphate (ATP) enzymatic activity in intestinal parasites, leading to a reduction in ATP production and retarded larval growth (Singh et al., 2022). NIC has various other effects, such as regulating the expression of various signal transduction pathway proteins, inducing exogenous and endogenous apoptosis, inhibiting protein expression related to tumor cell invasion and migration, decreasing mitochondrial membrane potential, increasing mitochondrial membrane permeability, and inhibiting cancer stem cells (Li et al., 2014; Xu et al., 2020a; Braga et al., 2021; Drozdzal et al., 2021; Kaushal et al., 2021; Jiang et al., 2022; Huang et al., 2023). In addition, NIC has inhibitory effects on various bacteria and viruses and can be used to treat diabetes, as well as cardiovascular, respiratory, and inflammatory diseases (Li et al., 2020). However, NIC has several disadvantages, including poor water solubility, low bioavailability, and potential for adverse reactions when regulating various signal transduction pathways (Wu et al., 2020). When administered systemically, it lacks ideal pharmacokinetic characteristics (Pacios et al., 2020). To enhance the therapeutic effects of NIC in clinical applications, it is necessary to modify the drug while retaining its chemical structure to increase its solubility, bioavailability, and targeting (Imperi et al., 2013; Barbosa et al., 2019; Lodagekar et al., 2019).

Poly(lactic-co-glycolic acid) (PLGA) is a biodegradable and biocompatible amphiphilic copolymer composed of poly(lactic acid) (PLA) and glycolic acid (GA) in varying ratios (Nkabinde et al., 2014). PLGA has good stability and excellent mechanical strength, and can degrade into water-soluble lactic acid and glycolic acid *in vivo*, which promotes drug release through surface erosion and other mechanisms (Vlachopoulos et al., 2022). PLGA is an FDA-approved high-performance polymer for drug delivery that effectively enhances the solubility, efficacy, and bioavailability of poorly soluble drugs (Jeon et al., 2020). However, the inherent hydrophobicity of PLGA hinders its interaction with cells, and hydrophilic surface modifications are required to improve its drug delivery efficiency (Grosso and de-Paz, 2021). Hyaluronic acid (HA) is a natural linear polysaccharide composed of d-glucuronic acid and N-acetylglucosamine linked by β-1,3 and β-1,4-glycosidic bonds. It is rich in carboxyl and hydroxyl groups, which make it highly hydrophilic. Additionally, HA has biocompatibility, biodegradability, safety, and is not immunogenic, making it a widely used hydrophilic drug delivery carrier in the medical field (Lee et al., 2020).

Microfluidics refers to the manipulation of small amounts of fluid in channels at the nanometre or micrometre level. Microfluidic technology can be used to adjust the composition, structure, and size of products by controlling the reactant concentration, flow rate, pore structure, and other factors. Microfluidic technology is widely used for nanoparticle preparation owing to its excellent flexibility and controllability (Tavakoli et al., 2022). Compared to traditional nanoparticle preparation methods, this technology can more precisely control the flow and dispersion of multiphase fluids in microscale spaces, enabling the precise regulation of the size, morphology, and structure of the composite particles. Additionally, the preparation system is closed to eliminate the environmental degradation of drugs and ensure sterility. In recent years, microfluidic technology has been widely applied in various fields, including drug delivery.

In this study, we employed microfluidic technology to prepare drug delivery carrier microspheres using NIC as a drug model and a double-layer fishbone chip as a template. PLGA and HA were used as drug delivery carriers. Through the sequential emulsification of two-phase solutions in the microchannels, a dispersed multiple emulsion was formed via layer-by-layer encapsulation. Using an ultrasound-assisted emulsion-evaporation method, a monodispersed load of chlorambucil was prepared in NIC@PLGA-HA composite nanoparticles (NIC@PLGA-HA NPs), which improved their dispersion stability in water. The drug release behavior of the drug-loaded microspheres and their inhibitory effects on the activity of Caco-2 cells were also studied.

## 2 Materials and methods

### 2.1 Materials

The following reagents and materials were used in this study. NIC (mass fraction 98%, Aladdin Reagent Co., Ltd.); PLGA (50:50 mass ratio of lactic acid to glycolic acid, Mw 24,000–38,000, 76,000–115,000, acid terminated, Aladdin Reagent Co., Ltd.); ethyl acetate (mass fraction 99.7%, Shanghai Macklin Biochemical Co., Ltd.); polyvinyl alcohol [PVA; type 105, alcoholysis degree: 98%–99% (mol/mol), Beijing WoKai Biological Technology Co., Ltd.]; Hyaluronate acid (HA, amine terminated, Mw 80,000–150,000, Shanghai Macklin Biochemical Co., Ltd.); 1xPhosphate Buffer solution (PBS) buffer (LangJiKe Technology Co., Ltd.); thiazolyl blue tetrazolium bromide (MTT; mass fraction 98%, Shanghai Macklin Biochemical Co., Ltd.); dimethyl sulfoxide (DMSO; molecular biology grade, mass fraction ≥99.9%, Aladdin Reagent Co., Ltd.); dialysis bag (retained relative molecular weight: 12,000–14,000).

### 2.2 Methods

#### 2.2.1 Transmission electron microscopy

Transmission electron microscopy (TEM) was used to observe the morphology of the prepared nanoparticles. About 10 μL of the sample was dropped onto a carbon-coated copper grid and air-dried under infrared radiation. The samples were observed at 200 kV.

#### 2.2.2 Fourier transform infrared spectroscopy

The NIC, PLGA, HA, and NIC@PLGA-HA microspheres were analysed separately using Fourier transform infrared spectrometer (FTIR) for full-band scanning over a range of 400–4,000 cm^−1^ and step size of 4 cm^−1^. The encapsulation of NIC was examined by analysing changes in the absorption peaks of various functional groups in the FTIR spectra.

#### 2.2.3 X-ray diffraction

X-ray diffraction (XRD) analysis of the nanoparticles was performed using a Rigaku Smart Lab powder X-ray diffractometer with Cu-Kα radiation over a 2θ range of 5°–40° at 37°C. The obtained data were plotted and analysed.

#### 2.2.4 Thermogravimetric analysis

The thermal stabilities of the NIC, NIC@PLGA, and NIC@PLGA-HA microspheres were analysed using a Diamond thermogravimetry/differential thermal analysis (TGA/DTG) instrument (PerkinElmer, United States). Freeze-dried nanoparticles (2 mg) were placed in an aluminum pan, and the weight-loss curves were recorded at a heating rate of 10°C/min in the range of 25°C–500°C under a nitrogen flow.

#### 2.2.5 Particle size and zeta potential

An appropriate amount of NIC@PLGA-HA microspheres was weighed and added to deionised water, and the mixture was sonicated until the nanoparticles were well dispersed. The particle size, polydispersity index (PDI), and zeta potential of the nanoparticles were measured using dynamic light scattering (DLS). The particle size (nm) of the nanoparticles was measured using DLS at a test temperature of 25°C ± 0.5°C, and the PDI of all samples was recorded to characterize the uniformity of the nanoparticles. The zeta potentials of the nanoparticles were determined using a laser Doppler anemometer. Three measurements were performed for each sample and the average values were calculated.

#### 2.2.6 Determination of encapsulation efficiency and drug loading

Chloramphenicol nitrate (2.8 mg) was accurately weighed and added to an ethanol solution to a volume of 50 mL. The solution was homogenised by sonication to prepare a 56 g/mL chloramphenicol nitrate–ethanol solution. The absorbance values were measured in the wavelength range of 200–500 nm, and the maximum absorption wavelength of chloramphenicol nitrate was determined to be 332 nm, after excluding interfering factors. Samples of 1, 2, 3, 5, 7, 8, and 9 mL of the 56 μg/mL chloramphenicol nitrate ethanol solution was diluted to a volume of 10 mL and mixed well. A UV spectrophotometer was used to determine the absorbance at 332 nm. The standard curve equation was y = 0.0594 × + 0.0419, with *R* ^2^ = 0.9966. The linear fit to the standard curve is considered good. A certain concentration of nanoparticles was added to 5 mL of ethanol, sonicated for 5 min (150 W, 100% amplitude), and then placed in a shaking incubator at 37°C and 100 rpm for 24 h. After 24 h, the solution was centrifuged at 14,000 rpm and 4°C for 15 min to collect the supernatant, and a UV spectrophotometer was used to measure the absorbance value at 332 nm. The encapsulation efficiency was calculated using EE = m_encap_/m_drug_ × 100% and the drug loading was calculated using DL = m_encap_/m_total_ × 100%, where m_encap_ is the mass of drug encapsulated in the nanoparticles (mg), m_drug_ is the total mass of drug added (mg), and m_total_ is the total mass of the drug-loaded nanoparticles (mg).

#### 2.2.7 *In-vitro* drug release

To simulate the *in-vivo* environment, PBS solutions with pH values of 2.0, 5.2, and 7.4 were chosen as the release media, according to the Chinese Pharmacopoeia (2015 edition). Chloramphenicol nitrate (1.6 mg) was weighed and added to 40 mL of the release medium, followed by ultrasonic mixing to prepare a chloramphenicol nitrate solution (40 g/mL). After measuring the absorbance in the wavelength range 200–500 nm, the maximum absorption wavelength of chloramphenicol nitrate was determined to be 370 nm excluding interfering factors. Aqueous solutions of chloramphenicol nitrate at concentrations of 40 μg/mL were prepared by taking aliquots of 1, 2, 3, 5, 7, 8, and 9 mL and diluting to 10 mL, followed by thorough mixing. The absorbance at 332 nm was measured using a UV spectrophotometer to obtain the standard curve equation: y = 0.0021x + 0.0102, with *R* ^2^ = 0.9973. The standard curve exhibited good linearity.

Certain amounts of NIC and NIC@PLGA-HA were weighed and suspended in 3 mL of the release media. These solutions were then placed in dialysis bags (molecular weight cutoff of 12,000 Da) that had been pretreated, and the bags were tightly sealed at both ends. The entire dialysis bag system was submerged in 100 mL of buffer medium. The mixture was then placed in a shaking incubator at 37°C and 100 rpm. Sampling at 0.5 h (*n* = 1), 1 h (*n* = 2), 2 h (*n* = 3), 4 h (*n* = 4), 6 h (*n* = 5), 8 h (*n* = 6), 12 h (*n* = 7), 24 h (*n* = 8), and 36 h (*n* = 9), 4 mL of dialysate was removed, and the same volume of blank release medium was added. The absorbance of each dialysate sample was measured at 370 nm using UV spectrophotometry and the corresponding sample concentration was calculated using the standard curve equation. The cumulative release percentage Q was calculated according to the following formula, based on the calculated Q for each group. An *in-vitro* release curve was plotted based on the calculated Q for each group.

$$Q=\frac{C_{n}V+\left(C_{n-1}V_{n-1}+C_{n-2}V_{n-2}+C_{n-3}V_{n-3}+\ldots +C_{1}V_{1}\right)}{m}\times 100\%$$ (1)

where C_n_ is the NIC concentration, obtained from the standard equation for the n_th_ sampling, n is time V is the total volume of the release medium, V_n-1_, V_n-2_, and V_n-3_ are the volumes of the medium removed during the (n-1)_th_, (n-2)_th_, and (n-3)_th_ samplings, respectively, and m is the mass of NIC in the dialysis bag. Finally, the *in-vitro* release curve was fitted to a mathematical model using data processing software.

#### 2.2.8 *In-vitro* cell proliferation study

The MTT assay was used to evaluate the proliferation activity of Caco-2 (human colon adenocarcinoma cells) cells treated with the prepared nanoparticles. Caco-2 cells in the logarithmic growth phase were seeded at a density of 7 × 10^4^ cells/mL, with 100 μL of cell suspension added to each well of a 96-well plate. The zero well contained only cell culture medium. The plate was incubated at 37°C in a 5% CO_2_ incubator for 24 h to allow the cells to adhere. Next, the culture medium was removed, the cells were washed with PBS, and culture medium containing NIC and NIC@PLGA-HA nanoparticles with equal drug concentrations (160 ng/mL, gradient dilution) were added to treat the cells. Culture medium containing equal concentrations of DMSO (≤0.5%) was added to the control wells. The plate was then incubated at 37°C in a 5% CO_2_ incubator for 48 h. After incubation, the culture medium was removed, the cells were washed with PBS, and then 90 μL of serum-free culture medium and 10 μL of MTT solution (5 mg/mL, 0.5% MTT) were added to each well and mixed. Then, the cells were incubated for another 4 h. After incubation, the culture medium was removed, 150 μL of DMSO was added to each well, and the plate was placed on a shaker at low speed for 10 min to fully dissolve the MTT crystals. The absorbance of each well was measured at 490 nm using an enzyme-linked immunosorbent assay reader. The average and standard deviations were calculated, and the relative cell activities of the experimental and blank groups were obtained using the following formula:

$$cell\;viability=\frac{\left(A_{E}-A_{B}\right)}{\left(A_{C}-A_{B}\right)}\times 100\%$$ (2)

Where, AE, AC and AB were defined as the absorbance of experimental samples, untreated samples and blank controls, respectively.

#### 2.2.9 Cell morphological analysis

The cell morphologies were observed using an inverted fluorescence microscope. Log-phase Caco-2 cells were seeded at a density of 7 × 10^4^ cells/mL, and 1 mL of the cell suspension was added to each well of a 12- or 24-well plate. Sterilized cell-carrier glass slides were placed at the bottom of each well to facilitate subsequent sealing operations. The blank well contained only culture medium. Then, the plate was incubated at 37°C with 5% CO_2_ for 24 h to allow the cells to adhere to the wall. Next, the culture medium was removed, and the cells were washed with PBS before adding 1 mL of culture medium containing an equal amount of (160 ng/mL, gradient dilution) NIC drug or NIC@PLGA-HA microspheres to the cells. The control well contained a culture medium with an equal concentration of DMSO (≤0.5%), and the plate was incubated for 48 h at 37°C in a 5% CO_2_ incubator. The culture medium was discarded, and the cells were fixed with 1 mL of 4% paraformaldehyde at room temperature for 15 min, followed by washing with PBS three times. A small amount of DAPI staining solution (10 g/mL) was added to each well to cover the sample, and the plate was incubated at 37°C for 3–5 min. Subsequently, the DAPI staining solution was removed and the cells were washed with PBS two or three times for 3–5 min each. A sealing liquid was used to reduce fluorescence quenching. The cell morphology was observed using a fluorescence microscope before or after sealing.

### 2.3 Preparation of NIC@PLGA-HA microspheres

NIC@PLGA-HA microspheres were prepared using a microfluidic system. A typical preparation process is as follows. First, 3.5 mg of NIC and 25.0 mg of PLGA were weighed, added to 5.0 mL of ethyl acetate, and then stirred until they were fully dissolved to obtain the oil phase. This oil phase was mixed with 10 mL of PVA aqueous solution (2 wt%), and 10 mg of HA was added to each 10 mL and stirred until the mixture was homogeneous. The mixture was injected into the microfluidic system using a micro-injection pump, and it was slowly further mixed through a microfluidic chip and slowly stirred for 10 min before collecting the mixed solution. Then, the emulsion was treated with 150 W ultrasound for 10 min (with cycles of 3 s on and 2 s off), placed on a magnetic stirrer, and stirred until the ethyl acetate completely evaporated and a solid precipitated. The excess liquid was removed by filtering and the solid was washed with deionized water, and placed in a dialysis bag for 4 h to remove free NIC. After 4 h, the solid was frozen overnight at −80°C and then freeze-dried for 48 h, followed by storage at 4°C.

## 3 Results and discussion

The ideal size of drug carrier nanospheres is 200–600 nm, which allows for better targeting when administered subcutaneously or intraperitoneally. Furthermore, is the zeta potential of the drug-loaded nanospheres exceeds 15 mV, the repulsive force between particles is strong enough to stabilize the particles in solution, meeting the stability requirements. Using PBS (pH = 7.4) as the dispersing medium, DLS was used to determine that the average particle size of chlorambucil in PBS was 2533.7 ± 235.2 nm, with a PDI of 0.541 ± 0.117 and zeta potential of −22.9 ± 1.9 mV. However, the particle size of the drug-loaded NIC@PLGA-HA composite nanospheres prepared using microfluidic technology was 286–583 nm, and the zeta potential reached −25.4 ± 0.41 mV, which is ideal for drug-delivery nanospheres (Table 1).

**TABLE 1:** Drug loading rate, hydrodynamic diameter (nm), polydispersity index (PDI) and Zeta potential (mV) of NIC@PLGA-HA nanospheres (NPH NPs).

NO.	Time/min	NIC/mg	PVA/wt%	Drug loading rate/%	Diameter/nm	Zeta potential/mV
NPH1	5	2.5	1.0	0.96	498.1 ± 12.3	−7.3 ± 0.00
NPH2	10	2.5	1.0	0.76	530.1 ± 13.3	−20.7 ± 1.00
NPH3	15	2.5	1.0	0.53	482.5 ± 21.9	−21.9 ± 6.30
NPH4	10	2.5	1.0	2.31	286.8 ± 5.1	−2.0 ± 0.10
NPH5	10	3.0	1.0	5.18	519.2 ± 64.6	−12.5 ± 0.30
NPH6	10	3.5	0.8	5.58	545.4 ± 15.4	−23.0 ± 0.62
NPH7	10	3.5	1.0	3.04	583.1 ± 143.4	−23.3 ± 1.64
NPH8	10	3.5	1.2	8.70	442.0 ± 18.8	−25.4 ± 0.41

Note: The amount of PLGA used is 25.0 mg and the amount of HA used is 10.0 mg.

This indicates that after loading the drug into the PLGA-HA nanospheres, the hydrophobic and hydrophilic structural units of the polymer modification layer can be adjusted to stably disperse the drug in PBS and obtain drug-loaded nanospheres of suitable size, which have the potential for targeted drug delivery. During the preparation process, the ultrasonic treatment did not significantly affect the size of the drug-loaded nanospheres, but it did significantly affect the zeta potential. For an ultrasound treatment time of 10 or 15 min, the absolute value of the zeta potential exceeded 15 mV, and the resulting drug-loaded nanospheres were more stable in PBS. Increasing the concentration of the organic-phase drug increased the size of the drug-loaded nanospheres. However, even when the concentration of the drug was increased to 3.5 mg, the size of the drug-loaded nanospheres remained below 600 nm, and the drug loading of the resulting NIC@PLGA-HA composite nanospheres also increased. A higher drug loading is beneficial for improving drug bioavailability. In addition, the concentration of PVA had the most significant effect on the drug-loading amount and particle size of the drug-loaded nanospheres. When the concentration of PVA reached 1.2%, the drug loading was the highest, the average particle size was 442.0 ± 18.8 nm, and the absolute value of the zeta potential increased to −25.4 ± 0.41 nm. It is speculated that an appropriate emulsifier concentration is conducive to the dispersion of droplets in the microfluidic channel to form a stable microreaction field to enable the effective loading of hydrophobic drugs. Note that 25.0 mg of PLGA and 10.0 mg of HA were used.

**FIGURE 1:**
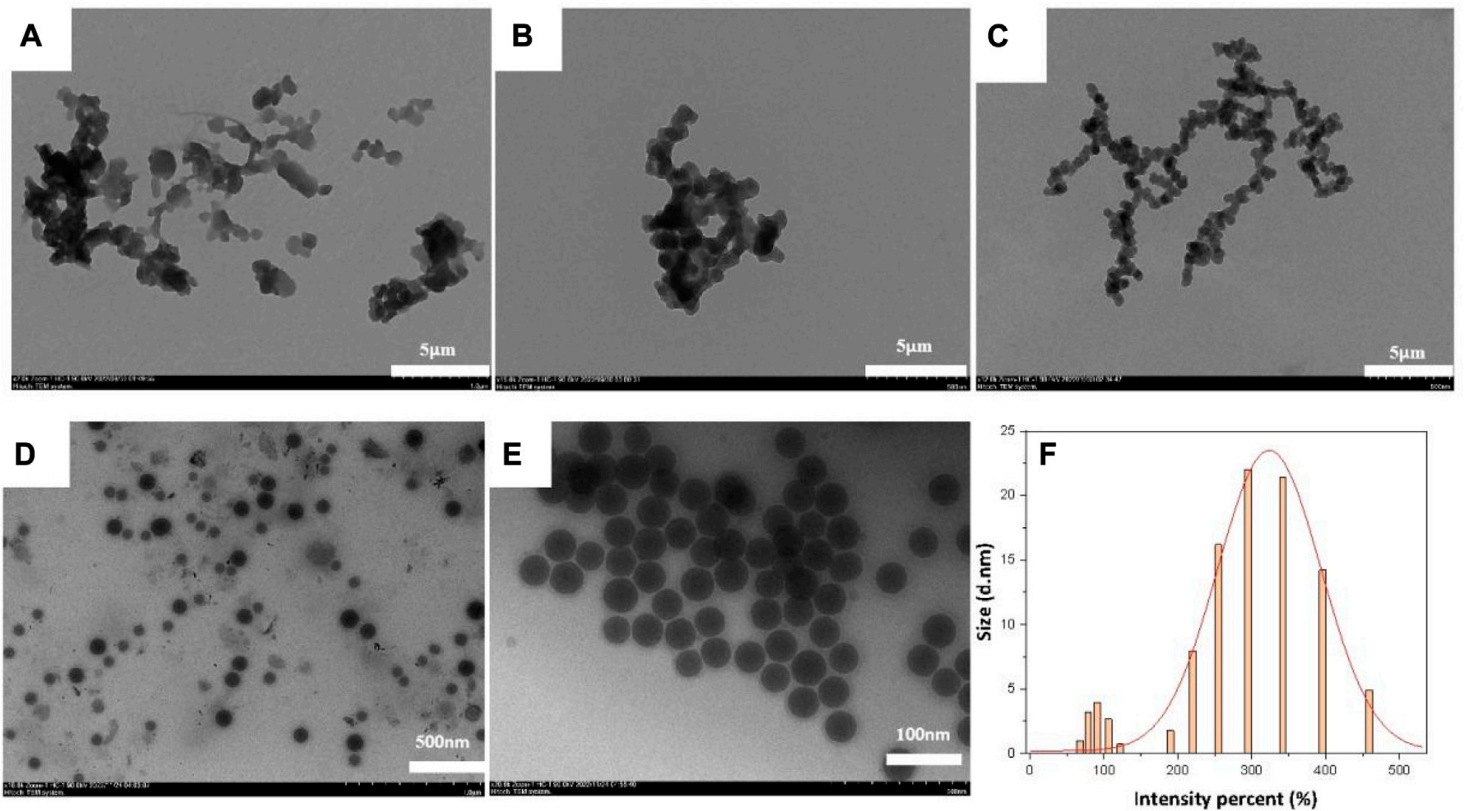
TEM images of NIC@PLGA-HA NPs: **(A)** NPH6, **(B)** NPH7, **(C,E)** NPH8, **(D)** NPH4 and **(F)**. The size distribution of NPH8 measured by DLS.

The main factors influencing the morphology of the NIC@PLGA-HA nanospheres were the concentration of the organic-phase drug and the ultrasound treatment time. When the drug loading was increased to 3.5 mg and the ultrasound time was 10 min, the resulting nanospheres maintained a spherical shape that is more conducive to drug release, with an average particle size of approximately 500 nm and a relatively uniform and smooth surface. The PVA concentration influenced the aggregation of the nanospheres. Higher concentrations of PVA were beneficial for stabilizing the droplets during synthesis, thereby reducing the aggregation of drug-loaded nanospheres. These results were obtained using TEM, as shown in Figure 1.

**FIGURE 2:**
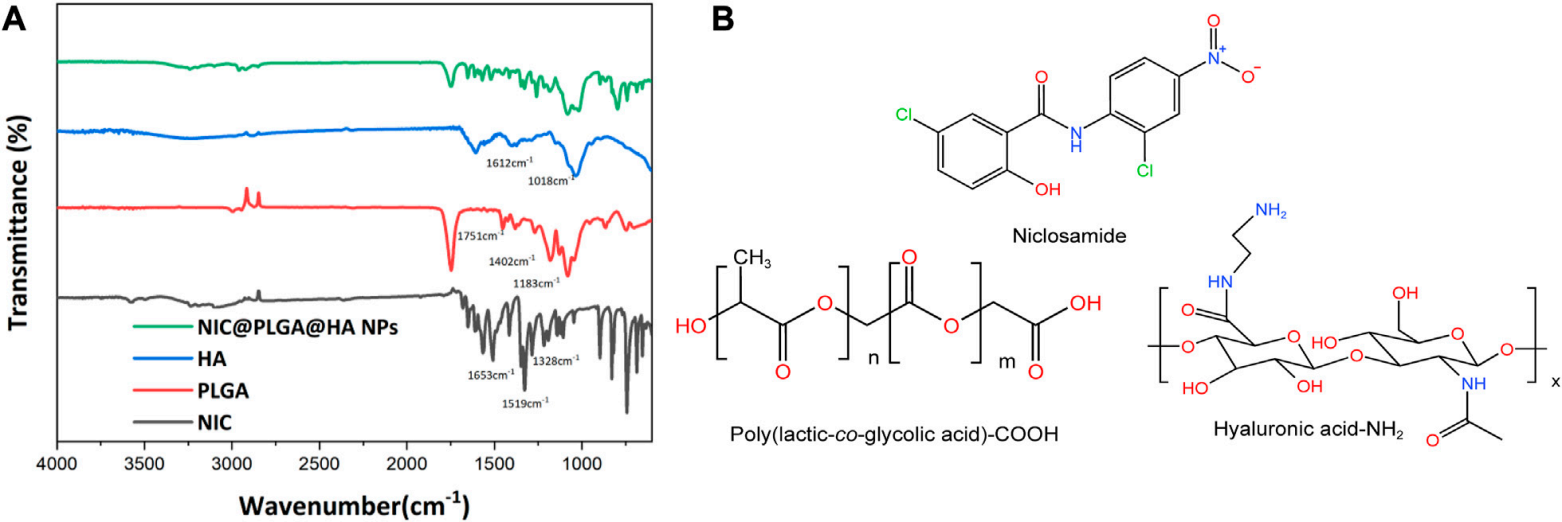
**(A)** FT-IR spectra, **(B)** Molecular structures of niclosamide, acid terminated PLGA and amine terminated HA.

In Figure 2, we show the infrared spectrum of the NIC@PLGA-HA nanospheres showed distinct characteristic absorption peaks for NIC at 1,328, 1,519, and 1,653 cm^−1^ correspond to the stretching vibration of –C=O on the amide bond, asymmetric stretching vibration of –NO_2_, and in-plane bending vibration of C–O in the phenolic hydroxyl groups, respectively (Chemical structures shown in Figure 2B). PLGA exhibits characteristic absorption peaks at 1,751, 1,452, and 1,183 cm^−1^ corresponding to stretching vibrations of C=O, C–H, and C–O–C, respectively. HA exhibits characteristic absorption peaks at 1,612, 1,414, and 1,018 cm^−1^ corresponding to the stretching vibrations of O–H and N–H, bending vibration of N–H, and stretching vibrations of C–O and C–N, respectively. The characteristic absorption peaks of the NIC@PLGA-HA nanospheres matched the above-mentioned characteristic peaks, indicating that the microspheres contained these structures, and the hyaluronic acid functionalized PLGA drug loaded nanoparticles were attained via physisorption upon HA and PLGA (Martins et al., 2018).

**FIGURE 3:**
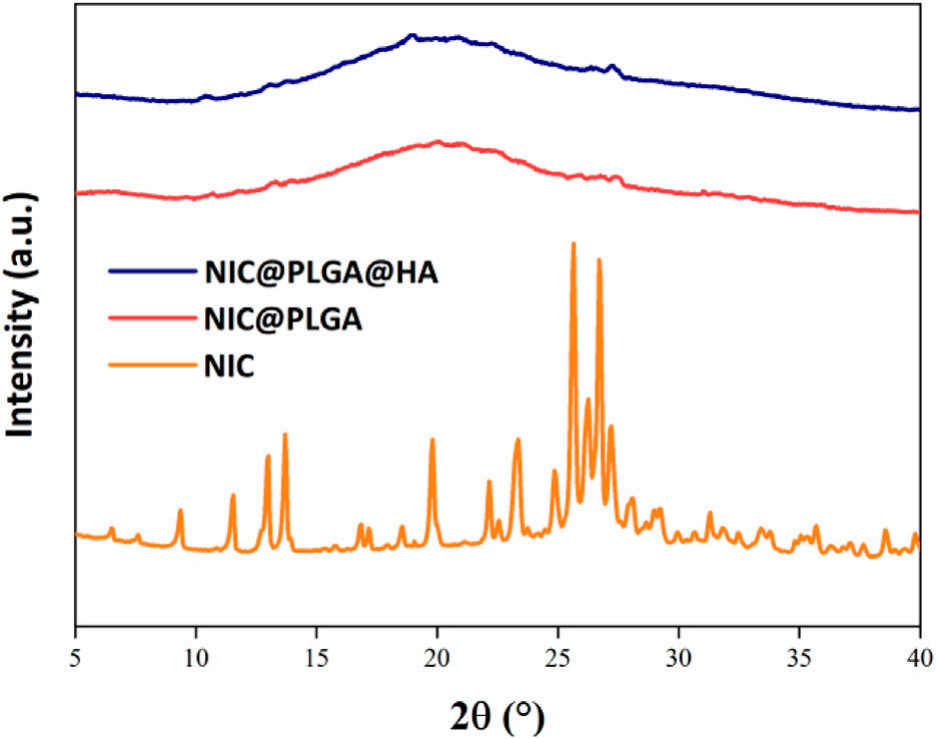
XRD patterns of NIC, PLGA and NIC@PLGA-HA NPs.

As shown in Figure 3, the XRD diffraction peaks of NIC are sharp, indicating its high crystallinity. In contrast, the XRD peaks of the drug-loaded NIC@PLGA-HA nanospheres were broad, which may have been due to the shielding effect of the PLGA-HA polymer layer that encapsulated the drug, resulting in a significant decrease in its apparent crystallinity.

**FIGURE 4:**
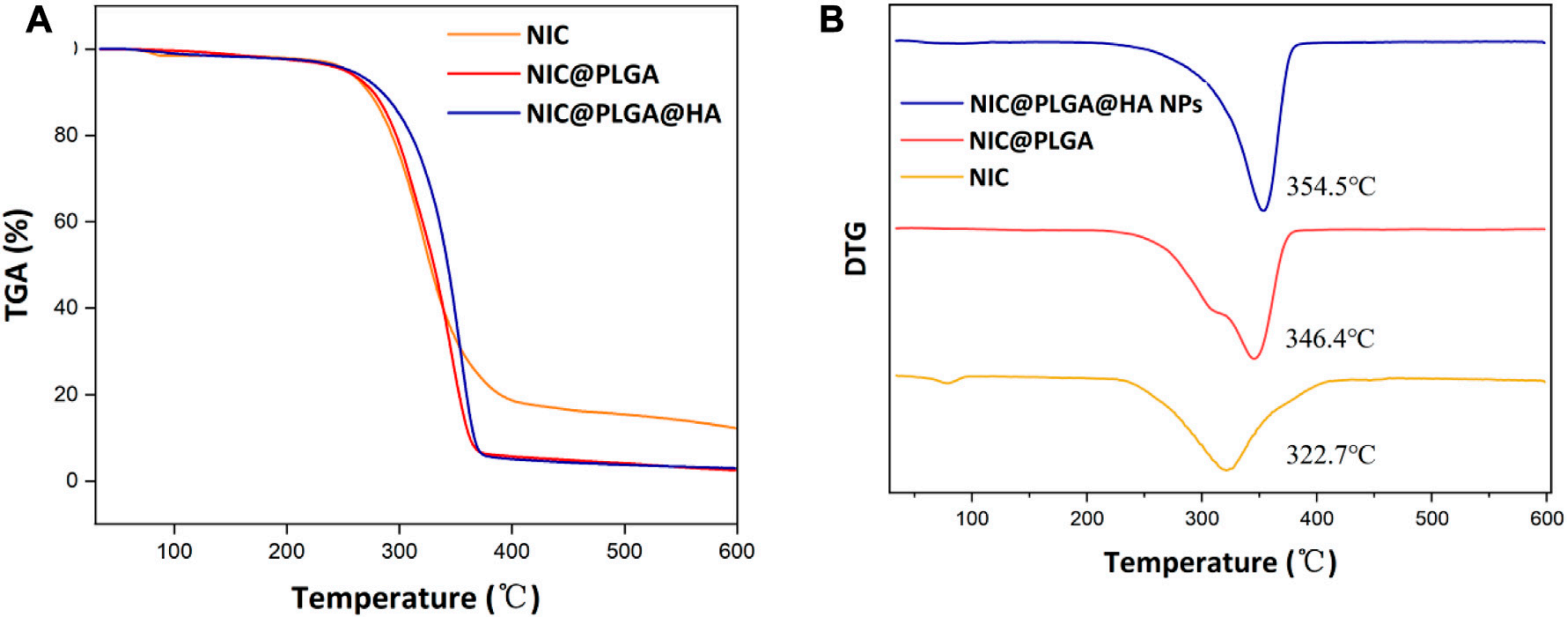
**(A)** TGA and **(B)** DTG curves of NIC, PLGA, NIC@PLGA-HA NPs.

The TGA results of the NIC@PLGA-HA nanospheres (Figure 4) show that NIC starts to decompose at 322.7°C, and the initial weight loss may be due to the volatilisation of water. The initial decomposition temperatures of NIC@PLGA and NIC@PLGA-HA nanospheres were 346.4°C and 354.5°C, respectively. At 400°C, the thermal weight loss of NIC was 81.5%, while those of NIC@PLGA and NIC@PLGA-HA nanospheres were 94.3% and 94.9%, respectively, mainly due to the thermal decomposition of some residual high molecular-weight polymers. The shift in the thermal decomposition peak may be due to stronger and more uniform intermolecular interactions between the loaded drug and the functionalized polymer coating, which results in a higher decomposition temperature of the drug-loaded microspheres (i.e., enhanced *in vivo* thermal stability).

**FIGURE 5:**
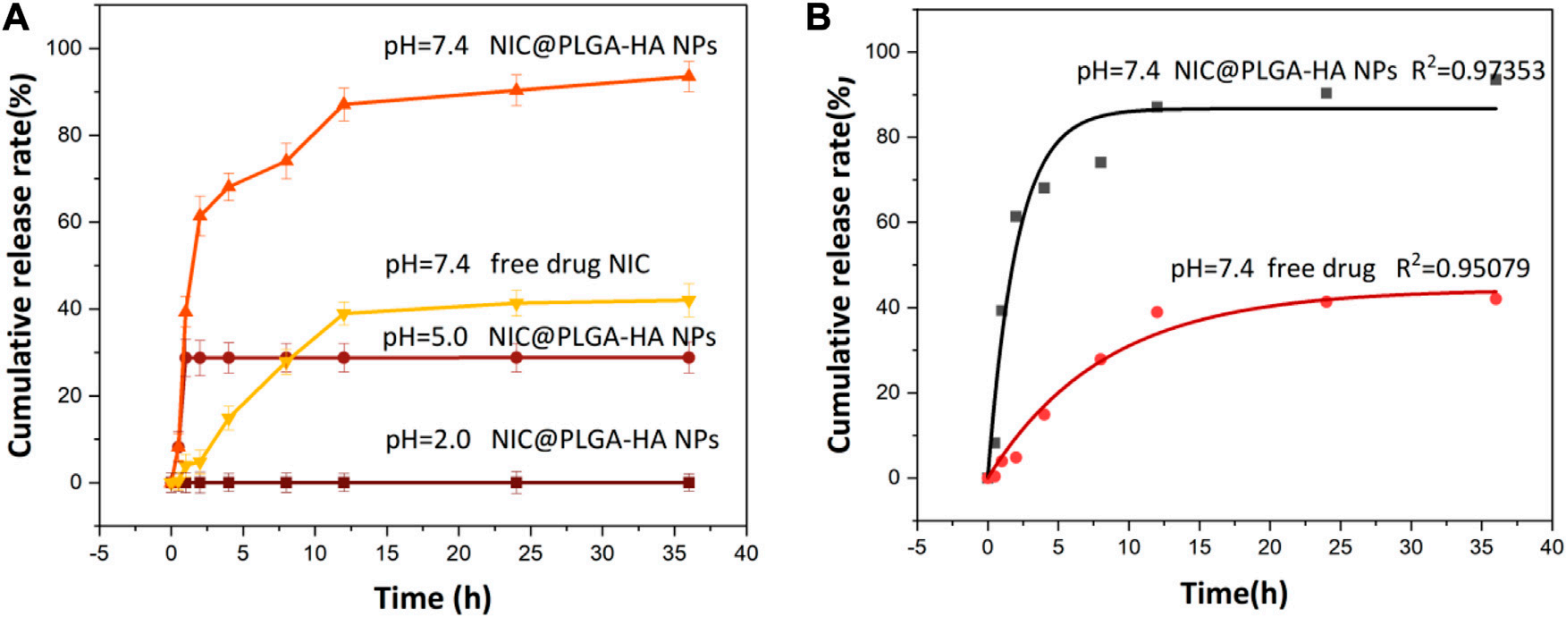
**(A)** Release profiles of NIC from drug loaded NIC@PLGA-HA NPs at different pH and **(B)** Release equation of free NIC and NIC from drug loaded NIC@PLGA-HA NPs at pH = 7.4.

The drug-release results of NIC@PLGA-HA nanoparticles in different pH media (Figure 5) show that there was no obvious release behavior of the nanoparticles within 36 h at pH 2.0. However, at pH 5.0, the NIC@PLGA-HA nanoparticles released some of the loaded NIC, with a release rate of 28.8% within 36 h. At pH 7.4, the drug release from the NIC@PLGA-HA nanoparticles were significant and efficient, and no obvious burst release phenomenon was observed. The release rate reached 93.5% within 36 h and the release kinetics were close to quasi-first-order release (Table 2). In contrast, the free NIC the release is only about 42.0% at pH 7.4 within 36 h. This result indicates the pH dependent nature of PLGA-HA coated NIC as expected due to higher solubility of PLGA-HA in neutral pH. This may be due to PLGA being degraded at pH 7.4 and releasing some of the drug, whereas HA remained stable. Therefore, the combined use of PLGA and HA can achieve a slow and sustained drug release. For pH values below 7.4, the degradation rate of PLGA is low, thereby reducing the drug release rate. Simultaneously, HA decomposes and releases the drugs. Before reaching the colon, the drug dosage forms must pass through the stomach (pH 1.5∼3.5), the duodenum (pH∼6), the intestine (pH 5.5∼6.8) and the caecum (6.8∼7.4) (McConnell et al., 2008). The pH-responsive colonic drug delivery to the intestine is that allow drug release when the pH increases as the drug dosage form enters the small intestine (Xu et al., 2020b). As prepared NIC@PLGA-HA nanospheres using PLGA-HA polymeric coatings that dissolve at these higher intestinal pH values (pH > 5.0∼7.4) should allow drug release upon reaching the colon to achieve colonic targeting drug delivery. The faster release of NIC in NIC@PLGA-HA NPs at pH 7.4 than pH 5.0 and 2.0 may be helpful in facilitating drug release in colony which usually have neutral pH. Therefore, the combined use of PLGA and HA under neutral conditions can achieve precise targeting colonic drug release.

**TABLE 2:** Fitting parameters of the drug-release kinetics of the NIC@PLGA-HA microsphere drugs at pH = 7.4.

Model	K	*R* ^2^
Zero-order kinetic equation	0.6092	0.7255
First-order kinetic equation	0.1518	0.9735
Higuchi equation	4.2796	0.9123
Korsmeyer-Peppas equation	5.9581	0.9263

In summary, the drug-release mechanisms of PLGA and HA may differ depending on the pH of the surrounding environment. At pH values lower than the pKa of the drug molecule, the drug molecules are protonated, which can increase their solubility in the surrounding medium. In slightly acidic environments, the use of hydrophobic PLGA with a higher molecular weight and acid-terminated groups as the inner encapsulation layer makes hydrolysis and degradation difficult, resulting in drug release. Under weakly acidic conditions, PLGA undergoes hydrolysis and degradation through its ester bonds, resulting in drug release. HA thereby releasing drugs via diffusion. At neutral pH, the degradation rate of PLGA is slower than that at acidic pH, and drug release is usually controlled by a combination of diffusion and polymer erosion. In contrast, HA expands slightly and degrades less under neutral pH conditions, and the drug release is mainly controlled by diffusion. Overall, the drug-release mechanisms of PLGA and HA at different pH values depend on the polymer properties, drug characteristics, and pH of the surrounding medium. By carefully selecting polymer and drug combinations, the drug-release mechanism can be optimized for specific applications, providing a good choice for drug delivery at different sites in the body.

To evaluate and explore the release mechanisms, zero-order, first-order, Higuchi, and Korsmeyer–Peppas models were used to fit the drug release kinetics.

Zero-order equation: M_t_/M_∞_ = K_0_t First-order equation: ln(1-M_t_/M_∞_) = −K_1t_

Higuchi equation: M_t_/M_∞_ = K_Ht_^1/2^

Korsmeyer-Peppas equation: M_t_/M_∞_ = K_t_^n^

Here, K_0_, K_1_, K_H_, and K are the drug-release rate constants of the zero-order, first-order, Higuchi, and Korsmeyer–Peppas models, respectively, and M_t_/M_∞_ is the drug release fraction at time t.

Table 2 shows the corresponding fitting parameters and correlation coefficient (*R* ^2^) values for the *in-vitro* drug release of NIC@PLGA-HA nanoparticles at pH 7.4. The release from the NIC@PLGA-HA microspheres was highly correlated with the quasi-first-order kinetic model, with *R* ^2^ = 0.9735. This model suggests that drug adsorption occurs primarily via chemical adsorption or physical adsorption. Due to slow diffusion of hydrophobic drug niclosamides, the drug release possibly mainly gets partitioned through the functionalized polymeric carrier PLGA-HA, possibly there had been some physical and chemical interactions occurring between the drug NIC functionalized polymeric carrier interfaces (Rezvantalab et al., 2018). Compared to free drugs, PLGA-based nanotherapeutics with intracellular triggered drug release have exhibited significantly enhanced antitumor efficacy on various solid tumors and drug resistant tumors.

**FIGURE 6:**
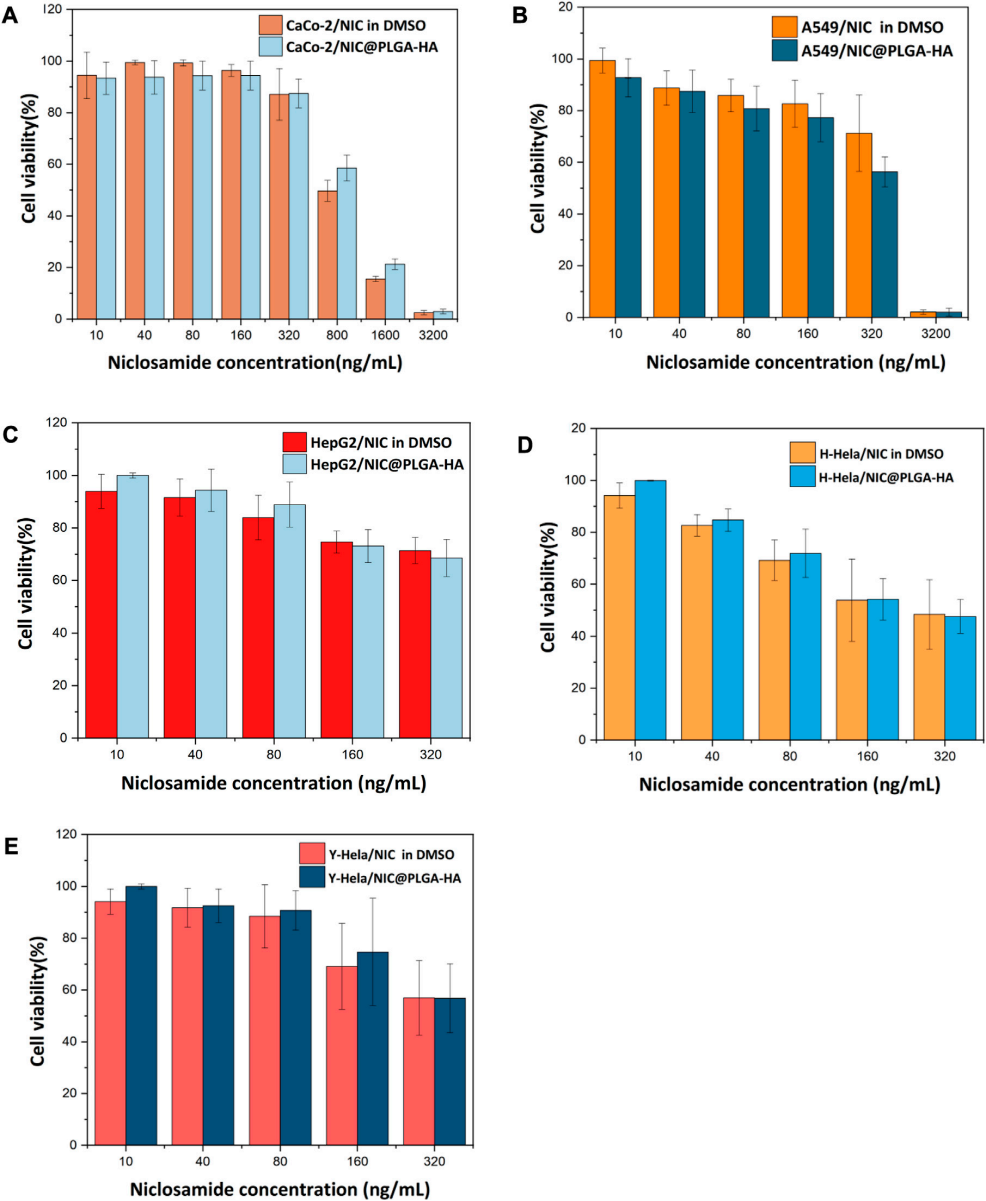
Effect of NIC and NIC@PLGA-HA microspheres on the proliferating ratio rate of Caco-2 cells **(A)**, A549 cells **(B)**, HepG2 cells **(C)**, H-Hela cells **(D)** and Y-Hela cells **(E)**.

**FIGURE 7:**
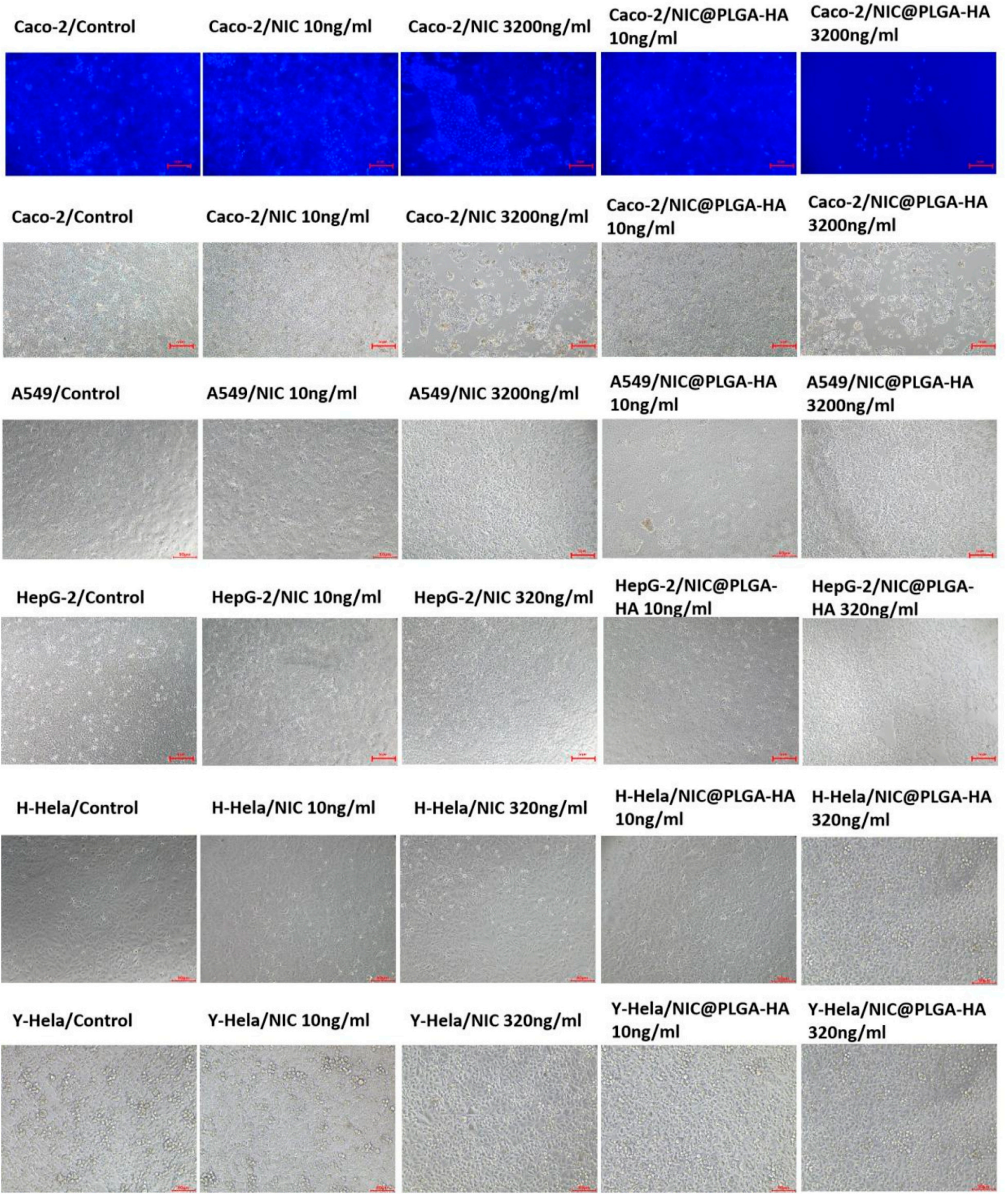
Effect of NIC and NIC@PLGA-HA microspheres on the metabolic activity Caco-2 cells and A549 cells, HepG2 cells, H-Hela cells, and Y-Hela cells.

The MTT assay results (Figure 6) showed that at a drug concentration of 200 ng/mL, the survival rate of Caco-2 cells was 100%, and there was no inhibitory effect on the proliferation of Caco-2 cells. Cell morphology analysis showed that, in contrast with the control group, after 48 h of exposure to 160 ng/mL NIC and 160 ng/mL NIC@PLGA-HA nanospheres, the number of Caco-2 cells remained high, and cell growth was not significantly affected (Figure 7). When the drug concentration was increased to 800 (cell viability 49.6%), 1,600 (cell viability 15.5%), and 3,200 ng/mL (cell viability 2.9%), the number of cells cultured with NIC gradually decreased, and the cell morphology changed. Meanwhile, NIC and NIC@PLGA-HA NPs exhibited similar cytotoxicity (cell viability 2.08%, 2.0%) on cancer A549 cells at the highest concentration of 3,200 ng/mL. Furthermore, NIC and NIC@PLGA-HA NPs exhibited cells cytotoxicity on cancer HepG2 (cell viability 71.3%, 68.5%), H-Hela (cell viability 48.4%, 47.6%) and Y-Hela cells (cell viability 56.9%, 56.8%) at the concentration of 320 ng/mL. These results confirmed that niclosamide and NIC@PLGA-HA NPs were specific towards cancer cells and decreased cell proliferation.

As the concentration increased, the degree of decrease in the number of cells and changes in cell morphology gradually increased. In particular, when the NIC concentration was 3,200 ng/mL, the Caco-2 cell morphology changed significantly, which is consistent with the results obtained from the MTT assay. In contrast, when the concentration of the NIC@PLGA-HA nanospheres was 3,200 ng/mL, the number of cultured cells decreased significantly, and the cells shrank and became spherical with a large area of cell shedding, which is consistent with the MTT assay results. This suggests that NIC has a better effect on inhibiting cancer cell growth through signaling pathways, but its hydrophobicity and changes in the crystal structure during the dissolution process affect its effectiveness in suppressing cancer cells.

Furthermore, the hydrophobic–hydrophilic polymer coating composed of PLGA and HA was conducive to the penetration of drug molecules, improving the stability of drugs in the system, prolonging the action time, and enhancing their inhibitory effects on tumor cells. In addition, this improved performance may be due to the carboxyl groups of glucuronic acid in the HA molecules, which can fully dissociate and form ion pairs with cations under physiological conditions or in appropriate pH environments. HA binds to CD44 receptors through its physical and chemical properties, such as the viscoelasticity and hydration, and promotes the aggregation of CD44 (Weng et al., 2022). CD44 is closely related to the growth, infiltration, and metastasis of tumors, thereby inducing cell apoptosis. Functional PLGA drug delivery systems can also improve the inhibition of the proliferation of Caco-2 cells by regulating their cell cycles (Luo et al., 2019; Moghaddam et al., 2021; Mohammadinejad et al., 2022). PLGA can release drugs *in vivo* and *in vitro* through mechanisms such as degradation into water-soluble lactic acid and glycolic acid, and surface erosion, thereby changing the cell environment and affecting the cell cycle. Therefore, the synergistic effects of PLGA-HA and NIC may increase the efficiency of drug-mediated inhibition of cell proliferation (Barbosa et al., 2019; Lu et al., 2020; Shen et al., 2020; Feltrin et al., 2022).

## 4 Conclusion

NIC@PLGA-HA microspheres were prepared using microfluidic technology and the loaded drug exhibited good biocompatibility with PLGA and HA. The average particle size of the microspheres was 442.0 ± 18.8 nm and their zeta potential was −25.4 ± 0.41 mV in water, indicating a stable dispersion. The drug-loading capacity of chlorambucil was 8.70%. The drug-loaded microspheres did not show significant sustained release behavior in simulated gastric fluid at pH 2.0. However, they exhibited sustained release behavior in simulated gastric protease and intestinal fluid at pH 7.4 and 5.0, respectively, and the drug-release kinetics followed quasi-first-order kinetics. At pH 5.0 and 7.4, the release rate of NIC from the NIC@PLGA-HA microspheres reached 28.8% and 93.5%, respectively, after 36 h of *in-vitro* drug release, which greatly improved the bioavailability of the drug for targeted delivery in the intestine. This PLGA-HA drug delivery system has the potential to be used as a carrier for oral administration and targeted therapy. Moreover, the results of *in-vitro* cell viability experiments showed that NIC and NIC@PLGA-HA microspheres inhibited the proliferation of Caco-2 cells, and the inhibitory effect of NIC@PLGA-HA microspheres on Caco-2 cells was more significant than the NIC drug at the same concentration.

## Data Availability

The raw data supporting the conclusion of this article will be made available by the authors, without undue reservation.
